# Emergency wounds treated with cyanoacrylate and long-term results in pediatrics: a series of cases; what are the advantages and boards?

**DOI:** 10.1186/1756-0500-2-132

**Published:** 2009-07-14

**Authors:** Betul Gulalp, Tamer Seyhan, Sonnur Gursoy, M Nur Altinors

**Affiliations:** 1 Department of Emergency Medicine, School of Medicine, Baskent University, Ankara, Turkey; 2 Plastic Surgery, School of Medicine, Baskent University, Ankara, Turkey

## Abstract

**Background:**

Ethyl-2-cyanoacrylate (ECA) is a tissue adhesive material applied to close superficial wounds. The aim of this study was to explore the benefits of cyanoacrylates in the emergency department in children with current application with regard to cost-effectiveness, satisfaction and long follow up.

**Findings:**

Patients were treated after assignment of the consent with an explanation by the relatives in a tertiary emergency department (ED), 2007.

The evaluation was based on different superficial wound repairs due to blunt trauma within a 2-hour time period (<6 hours), and small wounds (≤3 cm). These wounds were cleansed with serum sale and then dried with gauze. Wound repairs were observed for six months in order to observe the tissue changes. The patient's age, sex, indication, application time, pain score, cost, additional tending (if needed), complications, and cosmetic satisfaction were recorded.

A total of 9 patients were evaluated and followed for 6 months. Except for one, all children were treated without any serious complications. ECA was cost-effective, time-saving, and provided successful repair satisfaction by a blinded plastic surgeon and patient/parents.

**Conclusion:**

This report displayed the pediatric effective use of cyanoacrylates, even in non- traditional repairs in the emergency departments.

## Findings

Simple wound repair is a common treatment in the Emergency Department (ED). Cyanoacrylates are the current treatment used as tissue adhesives in repair of superficial lacerations. There are two biochemical forms, which are used generally for superficial repair, ethyl (ECA) and octyl-cyanoacrylates (OCA). Traditionally, superficial straight and generally small wounds can be repaired with only a piece of cyanoacrylate in different clinics of the hospitals (5,6,7,9).

Wounds in the mouth, non-straight wounds and/or those close to the joints can also be treated with simple supportive treatments at the ED. Besides, time, pain and the cost regarding the applications were the most important factors for the management. The aim was to display the usefulness of the superficial use of cyanoacrylates on children with different kinds of superficial wounds at the emergency department.

Nine patients were treated with a single thin-layer cyanoacrylate and made to wait for polymerization, each for one minute, then observed for 6 months, and applied between the period of January 1^st ^and December 31^st^, 2007.

The patients had presented to an emergency department at a university hospital. The patients enrolled were aged between 0 to 18 years with superficial traumatic wounds that needed repair without absorbable deep closed suture, less than 3 cm in length and less than 6 hours old.

Explanation was provided and signed consents were obtained prior to treatment. Specific exclusion criteria included situations where the patients had dirty wounds, or with a history of any keloid, and allergy to cyanoacrylates. The cases were invited to control after a day, three days, a week, and 6 months after ECA treatment.

All applications, decisions, controls (on the control of 1^st^, 3^rd^, 7^th ^days and 1 month after the treatment) and clinical managements of wound healing were performed by the same emergency physician only.

Age, sex, indication, application time, pain score using a five-point Likert scale, the cost, and if needed, the additional procedures, complication, cosmetic evaluation with visual analogue scale scores (VACS) using a 100-mm visual analogue scale (0 = worst scar at the left end and 100 = best scar at the right end) from photographs by a blinded plastic surgeon, a visual analogue scale (VAS) score by the families with 'non-satisfied' (at the 0-mm end) and 'most satisfied' (at the 100-mm end) after six months were evaluated [1].

These have been mentioned in Table 1 and the material's price as a single ECA format was 13.71$, propylene was 0.81$, local anasthetic (prilocaine) was 3.20$, plaster for short forearm was 3.23$ with exchange rate. The patients and the families determined the VAS score together according to the appearance of the healed wound. The mean age was 8.2 ± 5.4 (2–16). There were 5 (55.6%) males and 4 (44.4%) females. The mean total of the application time including the additional procedures was 4.7 ± 3.7 (2–12) minutes.

**Table 1:** Age, sex, indication, time, cost, additional treatment, complications, and cosmetic satisfaction have been demonstrated.

Patient	Age	Sex	Indi.∞	Mean repair time (minutes)	Cost ($)	Additional approach	Compli.Ω	Cosm.(VAS scores 06–10; plas.sur¶-parents ø)
1	2	F	Facial (Medial cheek-oblique-1.3 cm)	2+2	2 × 13.71	_	Opened same day	8.5-8
2	15	F	Dorsal phalanx (2^nd^, 3^rd^, 4^th ^phalanx- linear-0.8-1-1 cm)	2+10	13.71+3.23	Plaster	_	Missed at the end of 6 month
3	4	M	Facial (Lower corner chin- horizontal -non-straight-2.2 cm)	2+1+5	13.71+0.81+3.20	Anesthetic-single median suture	_	9–9.5
4	11	F	Facial (Medial forehead-vertical-1.6 cm)	2	13.71		Exuda	8–9
5	3	F	Facial (Medial chin-oblique-0.9 cm)	2	13.71	_	_	8–9.5
6	7	M	Facial (Lateral chin-oblique-1.8 cm)	2	13.71	_	_	8–9
7	16	M	Upper Lip (Corner-subflap-1.7 cm)	2	13.71	_	_	8–9.5
8	12	M	Mouth (Mucosal area+ lower lip's corner+lateral chin+upper lip-non-straight-1-1.1-0.4 cm)	2+1+5	13.71+0.81+3.20	Anesthetic-single median corner suture	_	8–9
9	4	M	Facial (Forehead-oblique 1.2 cm)	2	13.71	_	_	8–9.5

∞ Indication, Ω. Complication, ξ. Cosmesis. ¶Plastic surgeon.ø Patient and parents

**Figure 1:**
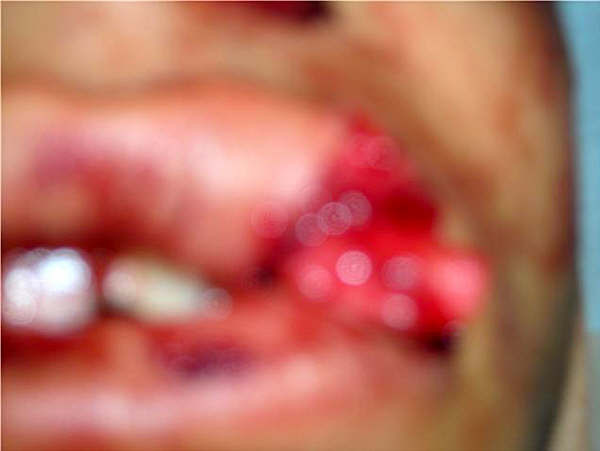
**A sub-flapped wound of superior corner of lip (7^th ^patient)**.

**Figure 2:**
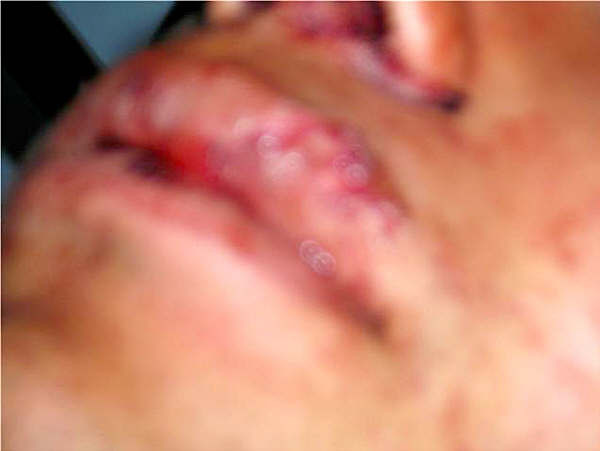
**Eftsoon of repair with ECA**.

The mean cost including the additional procedures was 16.5 ± 4.5$ (1$ = 1.240 YTL). The pain score was zero, except one that had 1 with a sensation of burning (a total score of 5) during application for a short transient time (<30 sn). VAS scores were 8,19 ± 0.37 (8–9), 95% CI 7,88–8,50 performed by the plastic surgeon, and 9,13 ± 0.52 (8–9,5) 95% CI 8,69–9,56 by the patients and the parents. The figures of the subflap of the lip of the 7^th ^patient before and after the closure can be seen in Figure 1,2,3. Figure 4 demonstrates a non-straight sub-chin wound after treatment.

**Figure 3:**
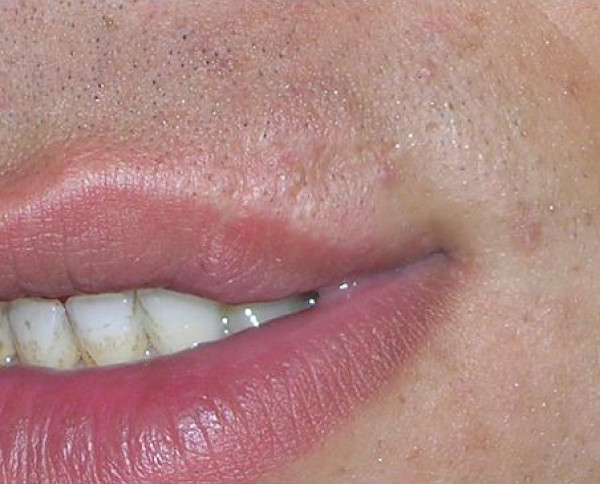
**A healed sub-flapped wound at the superior corner of the lip**.

**Figure 4:**
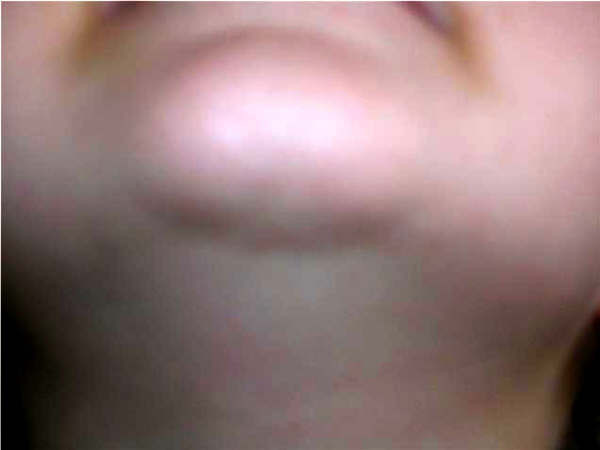
**A healed non-straight wound after a middle suture with cyanoacrylate treatment**. (3^rd ^patient).

The advantages of tissue adhesives have been reported due to them being cosmetic, cost-effective, satisfactory, the application being easily learnt, time-saving, and being less painful to sutures [1-8]. Minor blunt lacerations are common in ED. Cyanoacrylates are alternative materials to repair and ECA is traditionally used for superficial linear lacerations. Besides, this material can be used for various different parts of the body such as in mouth and extremity wounds [9-14]. The pain, the time spent, cost and satisfaction were the main factors which showed the best way for treatment of blunt minor lacerations in crowded and stressful clinics.

Pain was the one of the most serious reasons for anxiety in patients and parents. The other factors could be explained as a stressful environment and separation of the children from the parents. ECA was applied on small children while they lay on the lap of the parents and the parents lay on stretchers so as not to distribute their trusted feeling. There was generally no need for local anesthesia injection, which were essential for the traditional wound repair on children. It was used only on lacerations which were non-straight and/or closed to the corners.

Spauwen et al. reported that the results of lip closures using octyl-cyanoacrylate were successful [14]. Different conflicting conclusions were reported in the literature as the results of repair on edges with octyl-cyanoacrylate were less successful in especially younger patients than with sutures [7]. It was not generally used in the corner and/or with irregular lacerations by itself, we used only one suture with a thin circle of 4.0 propylene to close regularly in the corner and/or middle of the non-straight wounds, followed by application of ECA in our two patients. One of them had multiple superficial wounds around the mouth beginning from the inside of the mouth. Additionally, we used it on the distal extremity [9], with dorsal superficial multi-lacerations on metacarpal joints, and immobilized the extremity with a half-opened plaster for a week. There were previous studies that used cyanoacrylates in the mouth and lips by acting as an hemostatic agent of N-butyl-2-cyanoacrylate in the oral mucosa [10,11,15]. We repaired a sub-amputee lip to prevent tissue loss. Osmond et al. stated that the mean cost of tissue adhesives were $37.90 (Canadian dollars) [8], Karcioglu et al. showed that the costs of 15 patients who were treated with tissue adhesive were lower than $10 [2]. In our report, the mean cost of repair included the supported procedures, and not only the adhesives. Kharasch et al. reported a 19% complication rate associated with 2-octylcyanoacrylate use at 1-week follow-up [18]. In another study, gain of time, few wound complications and cosmetic satisfaction were reported with the use of tissue adhesive [12]. Zempsky et al. mentioned the time for closure of the wound as 3 minutes [3]. It was time-saving as the mean time spent for a patient was 4.6 minutes with additional procedures and generally did not require dressing in our report. Only one child, who was two years old, touched and removed the CEA on the same day. The application was repeated 9 hours after the first application. A dressing with gauze can be used to protect the repair in young children so that they do not touch their wounds. However, other children who were older than two years of age were careful about their wounds. There was only one serious complication. An 11-year-old girl, who complained about a minimal transient feeling like pain with a burning sensation while having ECA application, displayed an exuda occurring on the third day of treatment. The cyanoacrylate was removed, and then the culture sample was sent to the microbiology laboratory and the exuda was drained. Microorganism reproduction was not found in the results. It was concerned as a reactive allergic reaction due to cyanoacrylate. Cyanoacrylates have been occasionally reported as a cause of reactive allergic reactions due to local intoxication. Furthermore, recent reports have demonstrated that bacterial growth was inhibited with the effects of ECA. Cyanoacrylate was stated to have bacteriostatic effects and create a barrier protected from microorganisms in reports [13,16,17]. Quinn et al. reported the VAS scores results of 3 months as 60.6 mm [4]. All patients were followed-up for 6 months except one that mentioned no complaints and no local problems at the last period on phone-call. Zemsky et al. mentioned the cosmetic rating to be 37.0-23.6 mm for surgeons 1, and 50.6-16.4 mm for surgeons 2 after two-months (0 mm was the best scar) [3]. Karcioglu showed the distribution of patients' VAS scores that were 55.6% at 7, and 46.7% patients at 8 on the 90^th ^day (100 mm was the best scar) [2]. Holger et al. reported that the mean VACS values were 77.2 for reviewer A, 86.0 for reviewer B, and the VAS value was 82.0 for patients with octyl-cyanoacrylate at 9 to 12 months [1]. Our mean VACS and VAS values of the wounds after six months were 8.19 by the plastic surgeon, 9.13 by the parents, and cosmetic satisfaction was found to be appropriate by the surgeon and the families. There were some limitations in this report as it was a preliminary report, the number of the patients was low, it included different kinds of superficial lacerations, and there was no comparable group.

Consequently, it was cost-effective, in addition to multiple advantages for the patient and for the crowded clinics. ECA was useful in not only the traditional treatment of small wounds, but also in non-straight and different localizations with additional procedures if needed in the emergency department.

## Competing interests

The authors declare that they have no competing interests.

## Authors' contributions

BG designed, performed and wrote the study. TS evaluated the repairs. SG assisted the applications. MNA reviewed and supported the revisions.
